# Zinc improves neurological recovery by promoting angiogenesis via the astrocyte‐mediated HIF‐1α/VEGF signaling pathway in experimental stroke

**DOI:** 10.1111/cns.13918

**Published:** 2022-07-20

**Authors:** Yang Li, Tingting Ma, Xiaoyu Zhu, Mingqi Zhang, Liang Zhao, Peng Wang, Jia Liang

**Affiliations:** ^1^ Institution of Life Science Jinzhou Medical University Jinzhou China; ^2^ College of Pharmacy Jinzhou Medical University Jinzhou China; ^3^ Key Laboratory of Neurodegenerative Diseases of Liaoning Province, Department of Neurobiology Jinzhou Medical University Jinzhou China

**Keywords:** angiogenesis, astrocytes, HIF‐1α, stroke, VEGF‐A, VEGF‐R2, zinc

## Abstract

**Background:**

Ischemic stroke is a serious cerebrovascular disease with high morbidity and disability. Zinc accumulation has been shown to play a vital role in neuronal death and blood–brain barrier damage following ischemia in acute stage. However, almost nothing is known about whether zinc is involved in neurological recovery in ischemic prolonged period. This study investigates whether zinc promotes neurological recovery through astrocytes‐induced angiogenesis during ischemic repair phase.

**Methods:**

Sprague–Dawley rats were subjected to 2 h ischemia/14, 21, and 28 days reperfusion by middle cerebral artery occlusion, then administered ZnCl_2_ (10 mg/kg) via intraperitoneally daily from 7 days to tissue collection to observe brain tissue morphology, neurological function recovery by cortical width index, Adhesive removal test, and Forelimb placing test. Angiogenesis, astrocyte activation, and HIF‐1α/VEGF pathway were assessed via Western blot, immunofluorescence, and BrdU method in vivo and in vitro.

**Results:**

The results showed that zinc significantly alleviated brain atrophy and improved neurological function recovery during the cerebral ischemia repair stage. Zinc significantly increased the protein levels of HIF‐1α, VEGF‐A, and VEGF‐R2 in astrocytes, and promoted angiogenesis during cerebral ischemia repair. In vitro and in vivo studies confirmed that zinc promoted angiogenesis via the astrocyte‐mediated HIF‐1α/VEGF signaling pathway.

**Conclusions:**

Zinc significantly improves neurological function recovery during the cerebral ischemia repair stage, providing new evidence supporting zinc as a potential therapeutic target for ischemic stroke by promoting astrocyte induced angiogenesis.

## Graphical abstract

1. Zinc can alleviate cerebral ischemia atrophy and promote neurological function recovery during cerebral ischemia repair stage.
2. Zinc promoted angiogenesis via astrocyte‐mediated HIF‐1α/VEGF signaling pathway.
3. Zinc may serve as a potential therapeutic target for the treatment of functional.

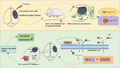

## 1 INTRODUCTION

The incidence of cerebrovascular disease is continuing to grow along with the aging global population, and the costs of long‐term care are still increasing due to the high disability rate.^1^ Stroke‐induced cerebral endothelial damage and inflammation impair endothelial function and thus increase cerebrovascular permeability and blood–brain barrier (BBB) leakage, leading to primary and secondary ischemic brain injury. Functional recovery is highly dependent on the effective restoration of blood supply to the damaged but viable peri‐infarct area.^2^ Therefore, extensive investigation has concentrated on poststroke vascularization with the aim of promoting the recovery process.

Zinc is an essential trace element that can be used as a neurotransmitter or neuromodulator and is essential for the growth and development of the brain. Zinc accumulates in neural synaptic vesicles in normal brain. In a microdialysis study, free zinc was released into the extracellular fluid during cerebral ischemia and after reperfusion.^3^ Accumulating zinc promotes injury of cerebral microvessels and trigger brain cell death. Recent studies showed that cytosolic zinc dramatically accumulated in neurons in the first a few hours of stroke, contributing to brain damage through promotion of neuronal cell death and BBB disruption.^4^, ^5^, ^6^ Till now, most studies of zinc in brain focused on the acute stage of cerebral ischemia, while the function and underlying mechanisms of zinc in ischemic recovery stage remain unclear. Zinc treatment in spinal cord injury model could regulate the expression of GPx4 and 4HNE through Nrf2/HO‐1 pathway to promote the survival of neurons, reduce spinal cord injury, and finally promote the recovery of neural function.^7^ Moreover, Zinc protoporphyrin attenuates whitematter injury after intracerebral hemorrhage.^8^ Therefore, zinc might be involved in promoting neurological function recovery during cerebral ischemia repair stage.

Astrocytes are considered by scientists to play an important role in maintaining the integrity and plasticity of neural function after cerebral ischemia. Neurotrophic factors, secreted by astrocytes, contribute to promoting brain remodeling, and improve long‐term neurological function recovery after stroke. In the repair phase of cerebral ischemia, proliferating reactive astrocytes migrate to the edge of the injury area.^9^, ^10^ Whether zinc can facilitate astrocyte activation during cerebral ischemia repair also needs to be investigated. Activated astrocytes are the main source of endothelin, vascular endothelial growth factor (VEGF), and angiopoietin 1, which are closely related to angiogenesis.^11^, ^12^ However, it is not clear whether zinc is involved in this process of angiogenesis. Hypoxia activates astrocytes to secrete hypoxia‐inducible factor 1α (HIF‐1α), the core regulator of angiogenesis in the repair phase of cerebral ischemia.^13^ HIF‐1α, a transcription factor, orchestrates angiogenesis by stimulating VEGF. VEGF, mainly derived from astrocytes, mediates angiogenesis through VEGF‐R2 in the central nervous system.^14^, ^15^, ^16^ In vitro studies have shown that zinc can regulate the expression of HIF‐1α in astrocytes after OGD/R treatment.^17^ Given this premise, we speculated that astrocytes‐mediated angiogenesis via HIF‐1α/VEGF pathway may be involved in the mechanism of zinc neural recovery in cerebral ischemic repair stage.

In this study, we investigated the therapeutic effect of systematic ZnCl_2_ on brain atrophy and neurological recovery after middle cerebral artery occlusion in rats. Our study demonstrated a critical role of zinc in neuroprotection during the cerebral ischemia repair stage.

## 2 MATERIALS AND METHODS

### 2.1 Rat focal cerebral ischemia/reperfusion model and drug administration

All animal experiments were approved by the Animal Care and Use Committee of Jinzhou Medical College and were conducted in accordance with the in vivo experiments (ARRIVE) guidelines for National Institutes of Health research.^18^ Male Sprague–Dawley rats (290–320 g) were anesthetized with 2% isoflurane and subjected to 2 h middle cerebral artery occlusion (MCAO) followed by 14, 21, or 28 days reperfusion using the suture occlusion model, fifty‐one rats that displayed circling to nonischemic side prior to reperfusion were considered to have a successful MCAO and included in this study: 9 rats were used for BrdU detection and 42 rats for the rest of the experiment, 2 rats without circling and one with subarachnoid hemorrhage were excluded.

In this experiment, the dose of ZnCl_2_ (10 mg/kg) was selected to investigate the effect of zinc on the cerebral ischemia repair stage. The concentration was selected based on others previous study.^19^, ^20^ ZnCl_2_ and NaCl were administered via intraperitoneal injection once a day from 7 to 28 days after reperfusion in a double‐blind manner. After that, the brain tissues were collected at 14, 21, and 28 days for further experiments.

### 2.2 Adhesive removal test

Sensorimotor deficits were assessed by investigators blinded to the study conditions before and 14, 21, and 28 days after ischemic stroke. Two pieces of sticky circular patch with an area of 63.62 mm^2^ were used as bilateral tactile stimuli attached at the distal‐radial region on the wrist of both forelimbs of rats. Normal rats managed to remove the paste under viscous stimulation, and the rats were familiar with the experimental environment before the experiment. The latency of adhesive removal was recorded during three trials per day for each forepaw. Before surgery, we trained rats continuously for 3 days, 3 times a day, with an interval of more than 10 min between the two experiments. We assessed rats per group for each time point.

### 2.3 Forelimb placing test

A square grid (3 × 3 cm) with a 1‐m long shelf was placed at a height of 1 m above the ground, and the rats were allowed to climb from one end of the shelf to the other within 1 min in the experiment. If the forelimb of the animal fell into the net during crawling, it was counted as 1 forelimb stepping error, and the total number of stepping errors on the contralateral forelimb of the experimental animal with cerebral ischemia within 1 min was counted.^21^ We assessed rats per group for each time point.

### 2.4 Measurement of cortical width index

After 14, 21, and 28 days of cerebral ischemia reperfusion, the brain was decapitated, and whole‐brain images were obtained with a digital camera. The width from the midpoint of the forebrain to the left and right sides was measured, and the ratio of the left and right sides width was the cortical width index.^22^

### 2.5 Proliferation measured by BrdU labeling

Bromodeoxyuridine (BrdU) labeling and immunostaining can be used to assess the spatial and temporal changes in cellular proliferation. Rats were administered BrdU (50 mg/kg) after MCAO for seven consecutive days. In normal mice, there were almost no BrdU+ cells in the cortex. Density of BrdU+ cells was exhibited in a temporally and spatially dependent manner.

### 2.6 Zinc detection by FluoZin‐3

Brain sections (16‐μm‐thick) were stained with the zinc‐selective fluorescent probe FluoZin‐3 (Invitrogen) to detect intracellular free zinc. To visualize zinc accumulation in cerebral tissue, sections were washed in PBS and incubated with FluoZin‐3 (2.5 μM) and DAPI (a nuclear specific marker) in the dark for 30 min. After washing in PBS, images were acquired with a fluorescence microscope and analyzed with ImageJ software. Three different areas in the peri‐infarct area from each rat were selected to count FluoZin‐3‐positive cells and analyze fluorescent intensity. The fluorescence intensity was assessed after subtraction of the background fluorescence intensity.

### 2.7 Immunofluorescence staining

Frozen 16‐μm‐thick brain sections were blocked with 5% normal goat serum for 1 h and then incubated with primary antibodies overnight. The primary antibodies were as follows: VEGF‐A (Abcam, Cat No. ab39250; 1:200); GFAP (Biolegend; Cat No. 837401, 1:500); HIF‐1α (R&D; Cat No. AF1935, 5 mg/ml) CD31 (Santa Cruz; Cat No. SC‐376764, 1:50), BrdU (Solarbio; Cat No. K006322 M, 1:50). The secondary antibody (1:200) was incubated for 2 h at room temperature. Fluozin‐3 (Invitrogen; Cat No. F24195) incubation for 30–45 min. Fluorescence images were obtained by a LEICA DMC6200 microscope.

### 2.8 Western blot

After ischemia reperfusion, the rats were anesthetized, the brain was decapitated, the corresponding protein was extracted, and western blotting was performed.^23^ The antibodies used in this study were VEGFA (Santa Cruz, Cat No. sc‐7269, 1:1000), VEGF‐R2 (Cell Signaling, Cat No. #9698, 1:1000), HIF‐1α (ZEN BIO, Cat No. 340462, 1:1000), GFAP (Biolegend, Cat No. 837401, 1:1000), and β‐actin (Cell Signaling, Cat No. #3700S, 1:2000), and the corresponding secondary antibodies (EARTH, Cat No. E030110‐01, 1:10,000), the membranes were incubated with the primary antibody overnight and secondary antibodies for 1 h. Images were captured by an Amersham Imager 600; analysis was performed using ImageJ software.

### 2.9 MTT assay

Cell viability was determined by using the thiazolium blue (MTT) colorimetric assay. The cells were placed in a 96‐well plate in the Con, OGD/R+SYP‐5 (MCE, Cat. No. HY‐100693) (HIF‐1α inhibitor) groups (2, 10, 50 μM) and incubated for 24 h to induce oxygen and glucose deprivation injury. After reoxygenation, the cells were washed with phosphate buffered saline (PBS) and then incubated in MTT (5 mg/ml) solution for 4 h. Then, 150 μl of dimethyl sulfoxide (DMSO) was added to each well, and the absorbance was measured at 490 nm to determine the cell viability with a microplate reader. The experiment was repeated 3 times.

### 2.10 Cell culture and oxygen and glucose deprivation (OGD) treatment

An optimal concentration was selected by MTT. To mimic ischemic‐like conditions in vitro, OGD treatment was performed on astrocyte cell lines (CTX TNA2). Astrocytes were plated in 6‐well plates and placed in a 37°C, 5% CO_2_ incubator for regular cultivation for 24 h. Astrocytes were randomly divided into control, OGD, and OGD/R+SYP‐5 (50 μM) groups. The control group was incubated for 7 h with glucose medium (DMEM/F12 glucose medium containing 1% penicillin–streptomycin solution) and replaced with complete medium (containing 10% FBS+1% penicillin–streptomycin solution DMEM/F12 with glucose medium). The OGD and OGD/R+SYP‐5 (50 μM) groups were added to glucose‐free medium (DMEM/F12 glucose‐free medium containing 1% penicillin–streptomycin solution) and incubated for 6 h in a hypoxic chamber at 37°C after 6 h of hypoxia. The complete medium containing 0 μM and 50 μM SYP‐5 was replaced with medium until the end of reoxygenation for 24 h.

### 2.11 Statistical analysis

Statistical analyses were performed using SPSS 19.0. Data are presented as the mean ± SD. One‐way analysis of variance (ANOVA) followed by LSD post hoc test were used to compare differences between multiple groups. *p* < 0.05 was defined as statistically significant.

## 3 RESULTS

### 3.1 Zinc alleviates brain atrophy and improves neurological function in the repair stage of ischemic stroke

**FIGURE 1:**
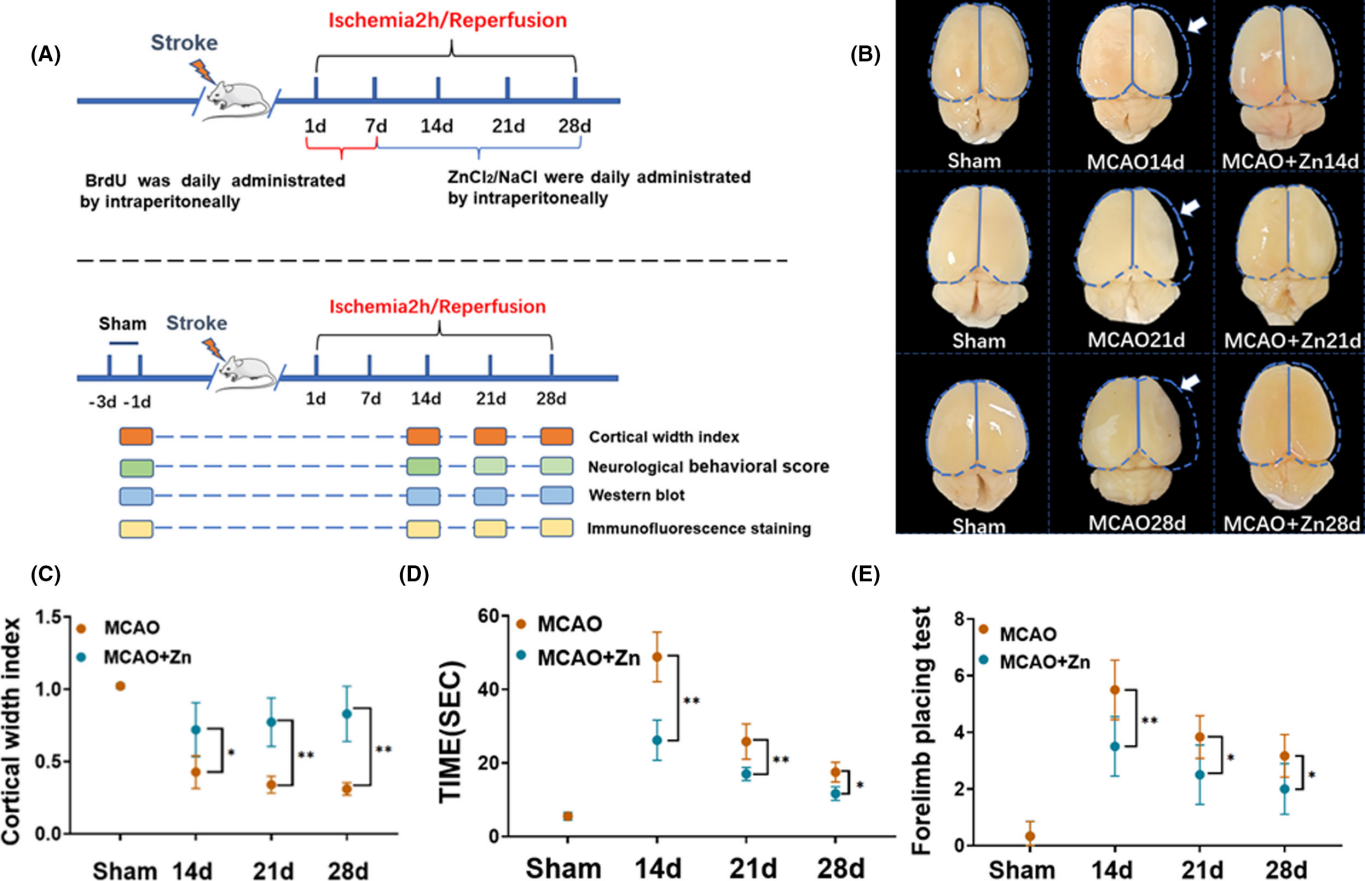
(A) At 2 h/7 days after ischemia–reperfusion, ZnCl_2_ was continuously intraperitoneally injected to 14, 21, and 28 days after ischemia–reperfusion, behavioral observations were performed on 14, 21, and 28 days after ischemia–reperfusion. (B,C) The cortical width index results showed that zinc reduces ischemia‐induced brain atrophy. (D) Tape removal test results showed that zinc reduces tape removal time and promotes nerve function recovery. (E) The results of the forelimb stepping experiment showed that zinc treatment decreased the number of forelimb stepping and suggested the promotion of nerve function recovery. *p < 0.05, **p < 0.005. Data are expressed as the mean ± SD (*n* = 6/group)

We first evaluated the function of ZnCl_2_ in cerebral ischemic damage in vivo, and the animal experimental design is depicted in Figure 1A. After 7 days of cerebral ischemia reperfusion, the rats were intraperitoneally injected with ZnCl_2_ (10 mg/kg) until 14, 21, and 28 days of ischemia reperfusion. Brain atrophy was assessed by measuring the cortical width index, as shown in Figure 1B,C. Intraperitoneal injection of ZnCl_2_ effectively reduced brain atrophy at 14, 21, and 28 days after MCAO. These results suggest that zinc may confer neuroprotection in the repair stage after ischemic stroke.

To further evaluate long‐term neurological recovery, we used the adhesive removal test and foot fault test to detect neurological function after treatment with ZnCl_2_ at 14, 21, and 28 days after MCAO. The time to remove the adhesive tape was significantly longer in the MCAO group than in the sham group. However, the adhesive removal time was significantly decreased after ZnCl_2_ treatment. The duration of the foot fault test was performed to assess forelimb and hindlimb function. We found that the number of forelimb steps was reduced after ZnCl_2_ treatment. These results demonstrated that zinc contributed to neurological recovery after ischemic stroke (Figure 1D,E).

### 3.2 Astrocytes are activated during the cerebral ischemia repair phase

**FIGURE 2:**
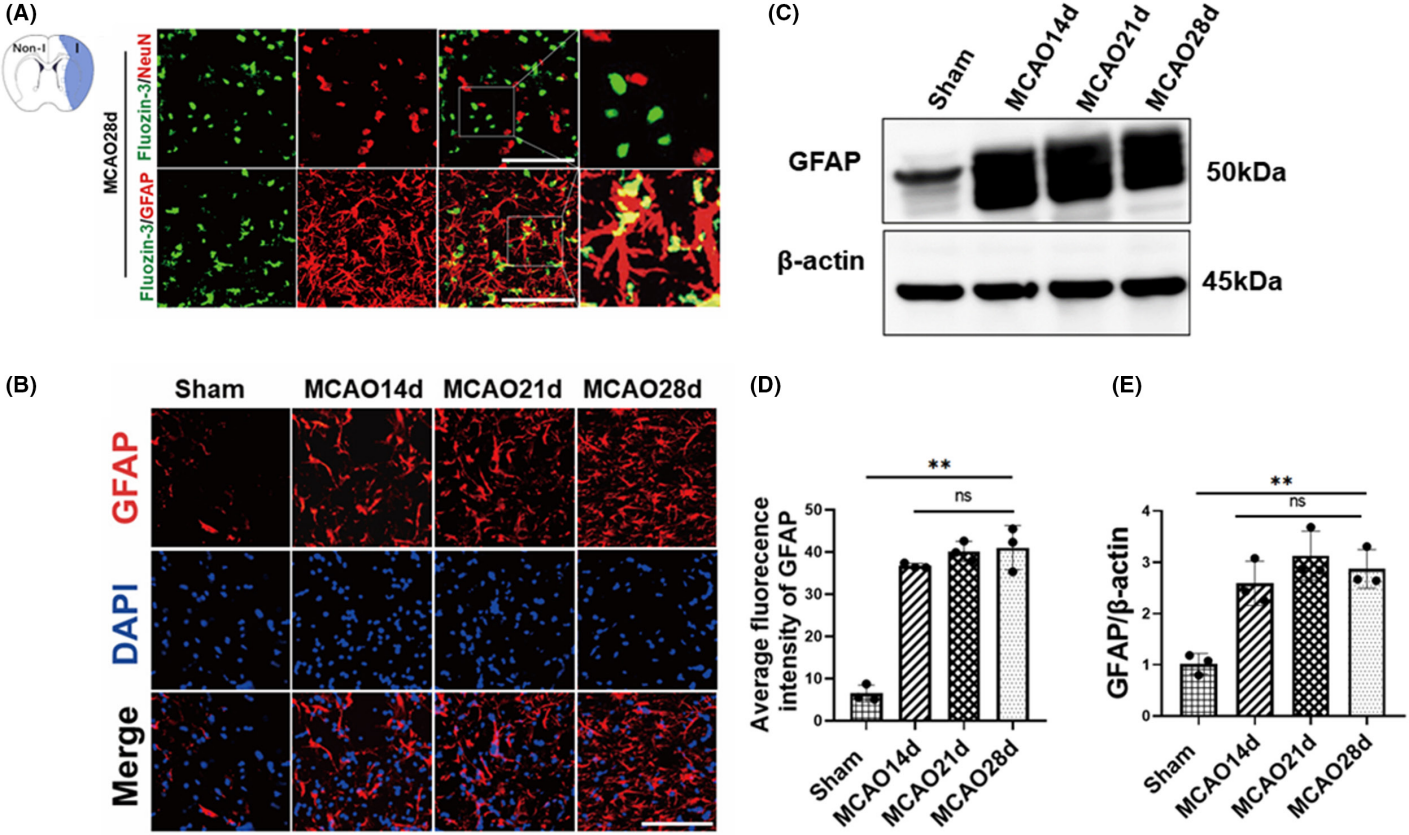
(A) Zinc accumulation in astrocytes and neurons brain slices was observed by costaining of zinc‐specific fluorescence indicator, FluoZin‐3 (green) with GFAP (red) and FluoZin‐3 (green) with NeuN (red) and nuclear marker (DAPI, blue). (B) Astrocytes are activated after cerebral ischemia, and their changes were observed by using an immunofluorescence staining approach 14, 21, and 28 days after cerebral ischemia. (C) GFAP protein expression was detected by western blotting at 14, 21, and 28 days after cerebral ischemia. (D) Fluorescence intensity was measured by costaining for a selective astrocyte marker (GFAP) and the nuclear specific marker DAPI. (E) Quantitative analysis of GFAP. (Scale bars: 20 μm) ns *p* > 0.05, **p < 0.005. Data are expressed as mean ± SD (*n* = 3/group)

To investigated the localization of zinc in peri‐infarct cortex during ischemic repair phase. We co‐stained brain slices with Fluozin‐3, NeuN, and GFAP. Double staining results showed that barely detectable Flouzin‐3‐positive cells in the neurons but more in astrocytes (Figure 2A). This finding indicated that zinc may help astrocyte to brain recovery. Next, we investigated the changes in astrocytes in ischemic and nonischemic hemispheres. We observed GFAP expression using an immunofluorescence staining approach (Figure 2B), while the nonischemic hemisphere revealed no trace of astrocyte activation, prolonged cerebral ischemia induced astrocyte activation, and GFAP fluorescence intensity increased significantly in ischemic hemispheres compared with nonischemic hemispheres. Most importantly, there was no significant difference in GFAP fluorescence intensity at 14, 21, and 28 days after ischemia reperfusion (Figure 2D). We investigated the protein expression levels of GFAP. The results showed that the protein expression level of GFAP was increased in the MCAO group compared with the sham group, but there was no significant difference in GFAP protein expression levels between 14, 21, and 28 days of ischemia reperfusion (Figure 2C,E). These results indicate the upregulation of GFAP after cerebral ischemia and astrocyte activation after ischemia. We chose 14 days after ischemia for next research.

### 3.3 Zinc facilitates the proliferation and activation of astrocytes during ischemia repair

**FIGURE 3:**
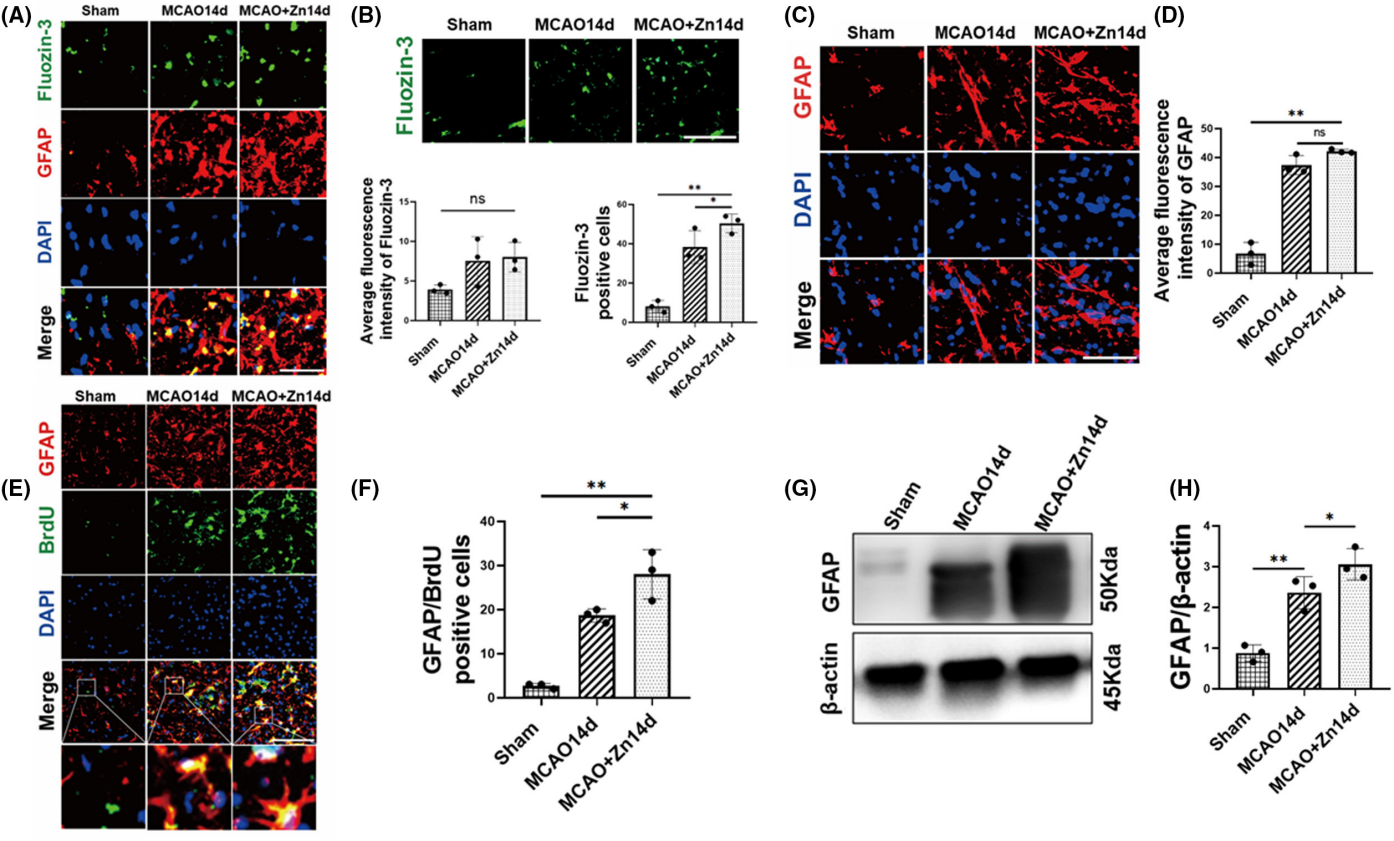
Zinc facilitates the activation of astrocytes in ischemic cerebral tissue. (A) Representative staining images for Fluozin‐3 (green), GFAP (red), and DAPI (blue). (B) Typical images of Fluozin‐3 staining in the ischemic hemispheres and nonischemic hemispheres or zinc‐treated rats. Quantitative analysis of the number of Fluozinc‐3‐positive cells that were counted in each rat. Quantitative analysis of the fluorescence intensity in Fluozin‐3‐positive cells in each group. (C) Costaining of GFAP (red) and DAPI (blue). (D) GFAP fluorescence intensity statistics. (E) Costaining of GFAP (red), BrdU (green), and DAPI (blue). (F) Quantitative analysis of the fluorescence intensity in GFAP/BrdU‐positive cells in each group. (G) The representative bands of GFAP using Western blot. (H) Quantitative analysis of GFAP. (Scale bars: 20 μm, field size:40 × 40 μm^2^), ns *p* > 0.05, *p < 0.05, **p < 0.005. Data are expressed as mean ± SD (*n* = 3/group)

Immunofluorescence staining showed that zinc acted on astrocytes after 14 days of ischemia–reperfusion, and after zinc treatment (Figure 3A), we analyzed the number of zinc‐positive cells and the change in average fluorescence intensity after cerebral ischemia (Figure 3B). The results suggested that the number of zinc‐positive cells in the ischemic hemisphere was significantly higher than that in the nonischemic hemisphere. Moreover, the number of zinc‐positive cells was significantly higher after ZnCl_2_ treatment than in the ischemic hemisphere. The average fluorescence intensity was analyzed, and the results showed that there was no significant change in the average fluorescence intensity of each group.

We further investigated whether the level of astrocytes could be regulated by zinc and used immunofluorescence staining to observe the activation and proliferation of astrocytes. After ischemia and hypoxia, astroglial cells change in size and morphology, and activated astrocytes are considered to contribute to functional recovery after stroke.^11^, ^12^, ^24^ Immunofluorescence results indicated that zinc promoted the activation of astrocytes after zinc treatment compared with the ischemic hemisphere. By analyzing the average fluorescence intensity, it was found that the fluorescence intensity of GFAP increased significantly in the ischemic hemisphere and after zinc treatment compared with the nonischemic hemisphere; however, there was no significant difference in fluorescence intensity between ischemic and nonischemic hemispheres after zinc treatment (Figure 3C,D). We also analyzed the number of GFAP/BrdU‐positive cells, and the results showed that compared with that in nonischemic hemispheres, the number of GFAP/BrdU‐positive cells was increased in ischemic hemispheres. Moreover, the number of GFAP/BrdU‐positive cells in the ischemic hemisphere increased significantly after zinc treatment compared with that in the ischemic hemisphere (Figure 3E,F). Western blot results showed that compared with the sham group, the level of GFAP protein in the MCAO group was increased, and the level of GFAP protein increased significantly after zinc treatment (Figure 3G,H). The above results suggested that zinc promoted the activation and proliferation of astrocytes in the stage of cerebral ischemia repair.

### 3.4 Zinc promotes angiogenesis during cerebral ischemia repair

**FIGURE 4:**
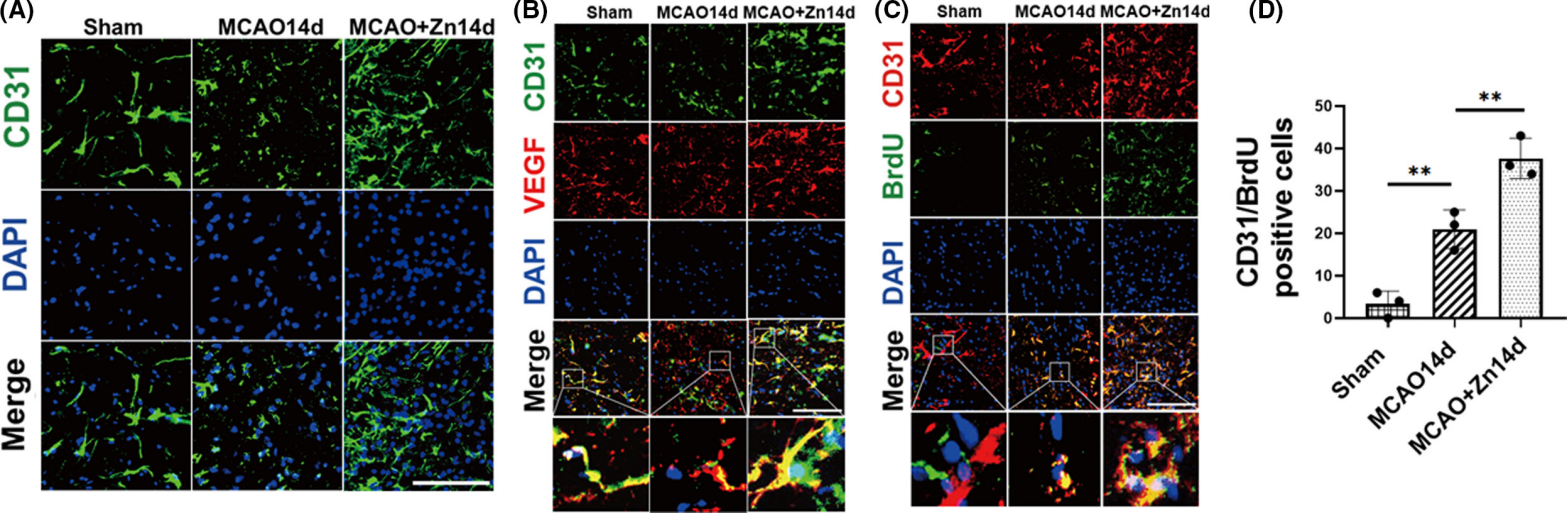
Zinc promotes angiogenesis during cerebral ischemia repair. (A) CD31 staining was performed to observe the changes in vascular morphology in ischemic hemispheres and nonischemic hemispheres or zinc‐treated rats. (B) Representative images of immunofluorescence staining for CD31 (green), VEGF (red), and DAPI (blue). (C) Representative images of immunofluorescence staining for CD31 (red), BrdU (green), and DAPI (blue). (D) Quantitative analysis of the number of CD31/BrdU‐positive cells in ischemic hemispheres and nonischemic hemispheres or zinc‐treated rats. (Scale bars: 20 μm, field size:40 × 40 μm^2^), **p < 0.005. Data are expressed as mean ± SD (*n* = 3/group)

Angiogenesis plays a protective role in the cerebral ischemia recovery stage. To investigate whether zinc promotes angiogenesis during cerebral ischemia repair, we studied the changes in vascular morphology during cerebral ischemia repair, and blood vessels were stained with CD31, which is a biomarker for endothelial cells. Blood vessels stain with spot or strip fluorescence. We found many microvessels in the peri‐infarct area (Figure 4A), suggesting that zinc may promote angiogenesis.

VEGF mediates angiogenesis through the vascular endothelium, as analyzed by immunofluorescence double staining for VEGF/CD31 (Figure 4B). To further confirm neo‐angiogenesis, the expression of neovascularization in the infarct boundary zone was analyzed by CD31/BrdU double immunostaining (Figure 4C). BrdU showed proliferating cells. Immunofluorescence staining showed that compared with the nonischemic hemisphere, the number of CD31/BrdU‐positive cells in the ischemic hemisphere was significantly increased, and the difference between the groups was statistically significant. The number of CD31/BrdU‐positive cells was significantly increased after ZnCl_2_ treatment compared with that in the ischemic hemisphere. The difference was statistically significant (Figure 4D), suggesting that zinc can promote angiogenesis during cerebral ischemia repair.

### 3.5 Zinc regulates ischemic cerebral injury via the astrocyte‐mediated HIF‐1α/VEGF signaling pathway

**FIGURE 5:**
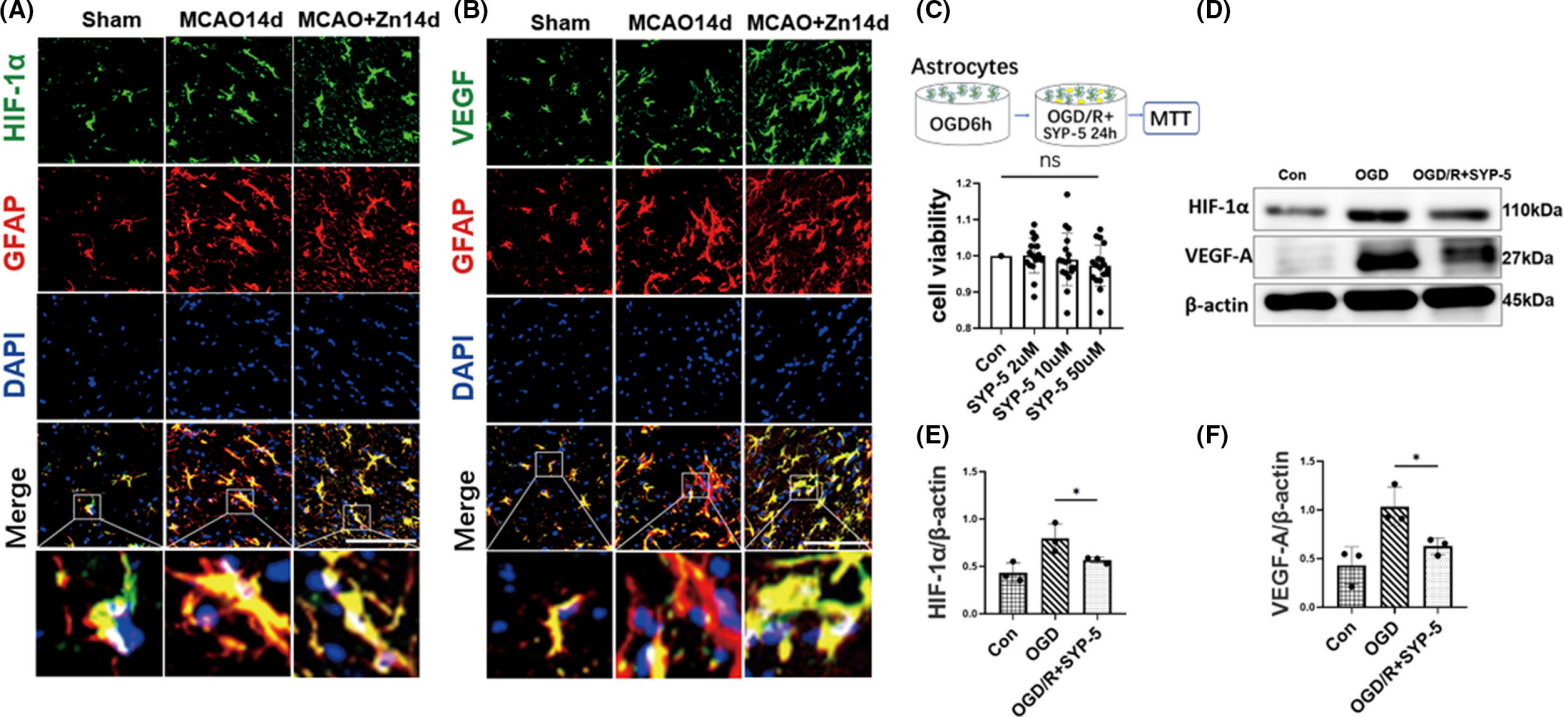
Zinc regulates ischemic cerebral injury via the astrocyte‐mediated HIF‐1α/VEGF signaling pathway. (A,B) Representative picture of GFAP on the HIF‐1α/VEGF pathway. Costaining of HIF‐1α (green), GFAP (red) and DAPI (blue) (Scale bars: 20 μm). (C) Cytotoxicity was detected by MTT. ns *p* > 0.05, (*n* = 18/group). (D) Western blot analysis of HIF‐1α and VEGF protein expression after the addition of a HIF‐1α inhibitor (SYP‐5). (E) Quantitative analysis of HIF‐1α. (F) Quantitative analysis of VEGF‐A. *p < 0.05. Data are expressed as mean ± SD (*n* = 3/group)

To explore the possible mechanism of zinc in promoting angiogenesis during repair stage following cerebral ischemic, we used immunofluorescence to double stain astrocytes marker (GFAP) with HIF‐1α and VEGF, as shown in Figure 5A,B, compared with sham group, more activated astrocytes co‐localized with HIF‐1α and VEGF in cytoplasm in MCAO14d and MCAO+Zn14d group. To confirm the astrocytes mediate the HIF‐1α/VEGF pathway, we design the cellular study using astrocyte cell line under OGD conditions (to mimic ischemic conditions). Astrocytes were treated with SYP‐5 (HIF‐1α inhibitor) at different concentrations of 2, 10, and 50 μM under 6 h OGD/24 h reoxygenation, and the cytotoxicity of SYP‐5 was detected by MTT assay. The MTT assay results showed that SYP‐5 had no significant cytotoxicity in astrocytes (Figure 5C). Accordingly, astrocytes were treated with a larger concentration of SYP‐5 (50 μM), and HIF‐1α and VEGF protein expression levels were detected by Western blotting. Both HIF‐1α and VEGF increased after OGD compared with the control group, while the protein levels of HIF‐1α and VEGF were significantly decreased after the addition of SYP‐5 (Figure 5D,F).

### 3.6 Zinc promotes angiogenesis via the HIF‐1α/VEGF signaling pathway

**FIGURE 6:**
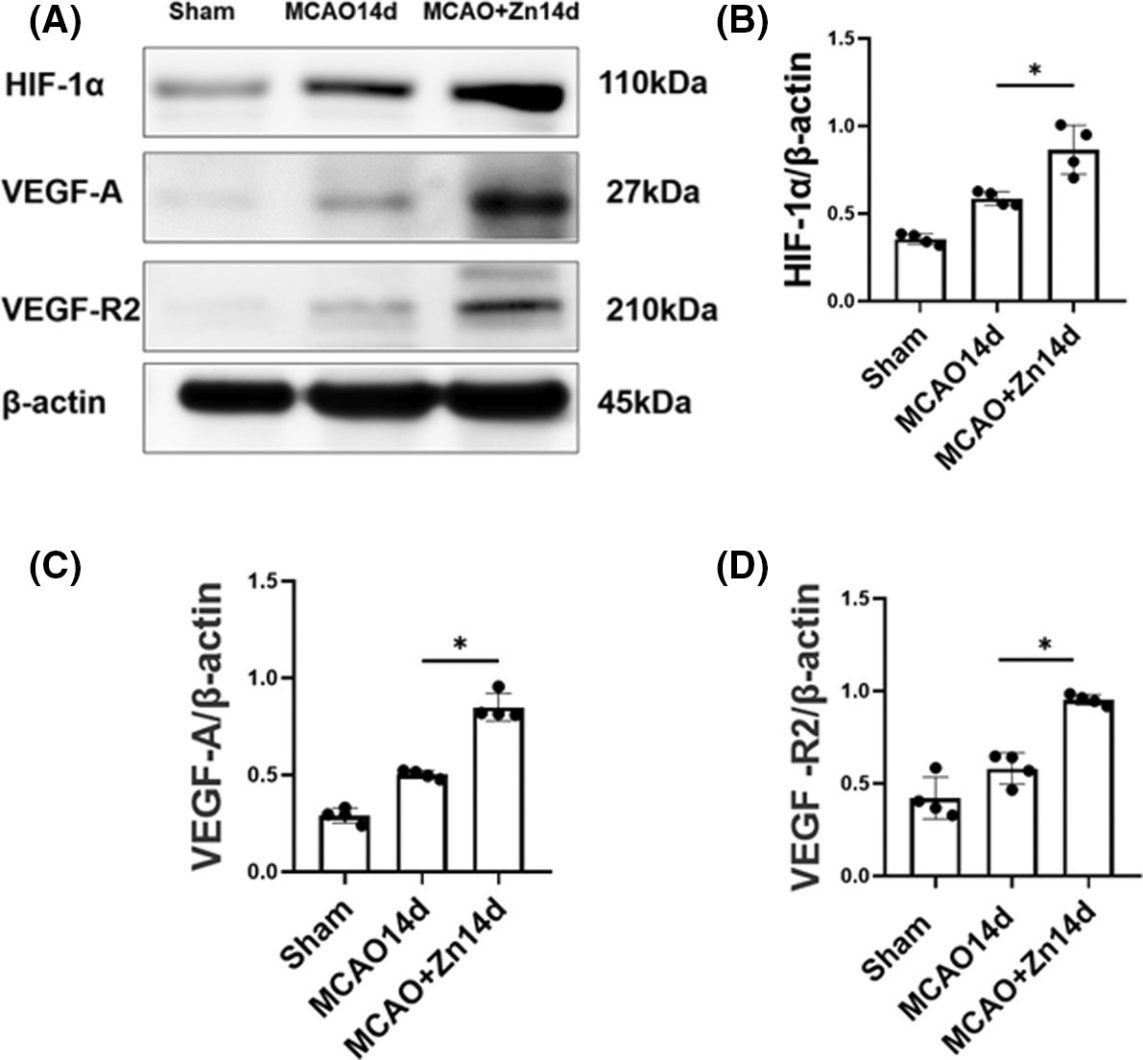
Zinc promotes angiogenesis via the HIF‐1α/VEGF signaling pathway. (A) Western blot analysis of VEGF‐A, HIF‐1α, and VEGF‐R2 levels in the sham group, MCAO group, and MCAO+Zn group. (B) Quantitative analysis of HIF‐1α. (C) Quantitative analysis of VEGF‐A. (D) Quantitative analysis of VEGF‐R2. *p < 0.05. Data are expressed as mean ± SD (*n* = 4/group)

HIF‐1α and VEGF mediate essential signaling in angiogenesis. We then detected the protein expression levels of VEGF‐A, HIF‐1α, and VEGF‐R2 by using Western blotting. As shown in ischemic stroke rats (Figure 6A–D), VEGF‐A, HIF‐1α, and VEGF‐R2 protein levels were increased at 14 days after MCAO compared to the sham group due to the endogenous protective effect of the body. After zinc treatment, the protein expression levels of VEGF‐A, HIF‐1α, and VEGF‐R2 were significantly increased compared to those in the MCAO group, providing experimental support that zinc promotes angiogenesis during ischemic repair by upregulating VEGF‐A, HIF‐1α, and VEGF‐R2. These results suggest that ZnCl_2_ can promote blood vessel regeneration during the cerebral ischemia repair phase.

## 4 DISCUSSION

This study investigated the protective effect of zinc in the ischemic stroke recovery stage. Our data demonstrated that zinc significantly reduced brain atrophy and improved neurologic deficits during the cerebral ischemia repair stage. We further explored the potential mechanism of these beneficial effects. We found that zinc promotes angiogenesis via the astrocyte‐mediated HIF‐1α/VEGF signaling pathway in experimental stroke. The findings from our experiments provide new insight into the role of zinc in cerebral injury after the ischemic stroke recovery stage.

Zinc is essential for normal cellular functions and serves a physiological and pathophysiological signaling role in the central nervous system.^25^ In acute phase of ischemic stroke, we and others have demonstrated that zinc is released from damaged neurons under ischemic conditions, causing a series of neuronal damages. Zinc was colocaliazed with neuron and microvessels.^5^ In our study, the fluorescence intensity of Fluozin‐3 in MCAO group is significantly enhanced compared with the Sham group (Figure 3A,B). However, zinc did not accumulate in neurons in repaired stage. Interestingly, zinc co‐located with activated astrocytes in repair stage after ischemia. Signals that mediate cell death during the acute stage of stroke might in fact promote repair during the recovery phase. The switch from injury to repair mediates this phenomenon highly dynamic in different cell types. In addition, accumulating studies have confirmed that in other central nervous system diseases, such as spinal cord injury and Alzheimer's disease, zinc can promote nerve repair through different mechanisms.^26^, ^27^ In our study, zinc treatment can significantly reduce brain atrophy and improve neurological function evaluation during repair, and this neuroprotective effect can last until 28 days. These results suggest that zinc plays different roles in different stages of cerebral ischemia, and the recovery of neurological function after brain injury may be regulated by regulating the balance of zinc in the brain.

Astrocytes, as very important nutritional support cells in the brain, contribute to angiogenesis, neurogenesis, synaptogenesis, and axonal remodeling and thereby promote neurological recovery. Activated astrocytes provide neuroprotection and contribute to neurorestoration.^28^, ^29^ Astrocytes are the most numerous and functional glial cells in the brain. After ischemia, astrocytes undergo dramatic changes in morphology associated with high GFAP expression and proliferation. We used the method of intraperitoneal injection of ZnCl_2_ at 7 days of ischemia–reperfusion until 14 days of ischemia–reperfusion to evaluate the activation of astrocytes. Findings from this study showed that zinc facilitated the activation and proliferation of astrocytes and promoted the repair of neurological function. Since reactive astrocytes have intimate contact with neurons and microvessels, we suspect that zinc promotes neuronal recovery in an indirect manner. Further research will explore the effects of zinc on neural remodeling to provide clues for new therapeutic avenues in the treatment of brain injury.

Angiogenesis refers to the germination of endothelial cells from existing blood vessels through proliferation and migration, forming new blood vessels and being regulated by a series of stimuli.^30^ Promoting angiogenesis can rapidly accelerate blood supply in the ischemic hemisphere, which is the premise of synaptic remodeling and neurogenesis. Our study found that 14 days after the cerebral ischemia repair period, in animals treated with zinc, the protein expression level of CD31 in the peri‐infarct area increased significantly, and the number of microvessels increased. In addition, we investigated the changes in vascular morphology during the repair phase of cerebral ischemia. We found many new small branches around the vessels in the peri‐infarct zone of the ischemic hemisphere after zinc treatment, so we suspected that zinc promoted the formation of new vessels, which is consistent with our hypothesis. Our findings indicated that zinc can promote angiogenesis in the repair stage, which provides a prerequisite for accelerating neurological function recovery.

Since recent studies indicated that hypoxia stimulates astrocytes to secrete HIF‐1α, exerts a neuroprotective effect and promotes neural functional recovery,^31^ for which VEGF is a downstream target gene that stimulates angiogenesis, maintains blood supply, and participates in neuroplasticity and brain neurological functional recovery,^32^ we further investigated whether zinc mediated the HIF‐1α/VEGF pathway during ischemic repair in vivo and in vitro. Our cellular experiment found that the expression of HIF‐1α is increased in astrocytes after OGD treatments. Using SYP‐5, a HIF‐1α inhibitor, can reduce the decrease in VEGF protein levels caused by OGD. HIF‐1α is a major regulator of angiogenesis and regulates the expression of many downstream target genes, such as VEGF, which is an important stimulator of angiogenesis and has an integral role in vascular development.^33^, ^34^ VEGF receptor 2 (VEGFR2) is the critical endothelial cell mediator of VEGF's proangiogenic activities and plays an indispensable role in developmental angiogenesis.^35^, ^36^ HIF‐1α is involved in hypoxia adaptation and angiogenesis through these genes.^37^ Thus, we investigated the effect of zinc on the levels of HIF‐1α, VEGF‐A, and VEGF‐R2 during the cerebral ischemia repair stage. Our data suggested that zinc promotes angiogenesis, alleviates brain atrophy, and promotes neurological recovery in experimental stroke through the astrocyte‐mediated HIF‐1α/VEGF signaling pathway.

In addition, our previous evidence in acute cerebral ischemia showed that zinc mainly acts on neurons and vasculature.^4^, ^38^ In this study on the repair period after cerebral ischemia, we found that zinc colocalized with astrocytes, but there was little or no colocalization with neurons (Figure 2A). These results might partially explain the different effects of zinc due to different cells in the acute stages and recovery stage of cerebral ischemia. Our study only detected angiogenesis after zinc treatment at 14 days as the time point for zinc to participate in neurological function recovery during cerebral ischemia repair. In future research, we need to further deepen the study of zinc on cerebral ischemic injury not only in time and space but also in other aspects of neurological repair, such as synapse formation and nerve regeneration.

## 5 CONCLUSIONS

In summary, our findings demonstrate that zinc promotes angiogenesis and protects brain tissue by regulating astrocyte HIF‐α/VEGF pathway during ischemic repair, reduces brain atrophy, and improves neurological function recovery. Regulation of zinc expression may be a feasible new target for the treatment of ischemic stroke.

## AUTHOR CONTRIBUTIONS

Jia Liang and Yang Li designed and supervised the study. Yang Li, Tingting Ma, and Xiaoyu Zhu performed the research and data analyses. Jia Liang, Yang Li, Xiaoyu Zhu, and Mingqi Zhang performed basic studies. Jia Liang and Peng Wang: concept and design of the study and review and editing of the manuscript. All authors approved the final manuscript.

## CONFLICT OF INTEREST

None.

## Data Availability

The data that support the findings of this study are available from the corresponding author upon reasonable request.
